# Speaking the Same Language: A Multi-Disciplinary QI Approach to Improving Handovers Across Acute Mental Health Wards

**DOI:** 10.1192/bjo.2026.11459

**Published:** 2026-06-30

**Authors:** Emily Pettifor, Ruwanka Uhanowita Marage, Ayebatonye Ajiteru

**Affiliations:** 1 Kent and Medway Mental Health NHS Trust, Dartford, United Kingdom; 2 Kent and Medway Mental Health NHS Trust, Maidstone, United Kingdom

## Abstract

**Aims:**

Effective clinical handover is essential for patient safety within mental health inpatient hospitals. However, concerns were raised at medical resident–senior forums highlightinginconsistent handover quality between ward staff and on-call medical doctors. Although handover policies existed, they did not clearly address interdisciplinary communication or clinical escalation pathways. This prompted a quality improvement (QI) project aiming to improve structure, clarity and consistency in handover practice. The aim was to improve quality and consistency of clinical handovers between ward staff and on-call doctors, and to implement a clearer, standardised interdisciplinary handover process.

**Methods:**

A mixed-methods baseline assessment was completed in 2024 involving questionnaires for nursing and medical teams, multidisciplinary process mapping, and root-cause analysis via fishbone methodology. Key issues identified included lack of structure and key information within handovers, unclear urgency or action required, and uncertainty around escalation routes if on-call phones are unanswered. Interventions selected through PICK chart analysis included: SBAR simulations delivered on pilot wards, SBAR prompt posters, embedding SBAR within junior doctor induction, developing a clinical escalation protocol with matrons, appointing locality SBAR champions (from multidisciplinary backgrounds) and trust-wide communication via screensavers and intranet SBAR resources. Questionnaire data was again collected in 2026.

**Results:**

Following implementation of the interventions, ward staff reported overall improvements in handover quality. Good–Excellent ratings for doctor-to-ward handovers increased from 53% to 75% and use of structured approaches by doctors increased from 44% to 50%. Staff understanding of patients following handover rose from 82% to 93%. Self-rated handover confidence (≥8/10) increased from 56% to 82%, with staff attributing this to training, experience and improved clarity of information. 93% of staff self-reported sometimes or always using handover structure. Ward managers on pilot simulation wardsreported improvements in regular SBAR use and staff confidence several months afterwards. Doctors reported fewer poor-quality handovers from wards, though improvements are still needed. The proportion reporting no structure to ward handovers reduced from 92% to 50% and 94% of doctors self-reported using a handover structure themselves. Further areas that still required improvement included variable on-call phone signal, and lack of clarity from some ward handovers.

**Conclusion:**

Co-designed interventions like SBAR training, visual prompting, clear escalation pathways and locality champions improved confidence, structure and standardisation in interdisciplinary handovers. Sustaining these improvements will require continued reinforcement, wider simulation rollout and senior leadership support, reflecting the challenge of changing communication culture. Following these results, the inpatient handover policy has been updated and distributed trust-wide.

